# The rocky road of 55 years of change in the relationship of cardiovascular risk factors to cognition

**DOI:** 10.21203/rs.3.rs-2557208/v1

**Published:** 2023-02-15

**Authors:** Osorio Meirelles, Anthony Arnette, Vilmundur Gudnason, L. Launer

**Affiliations:** National Institute on Aging Intramural Research Program; National Institute on Aging Intramural Research Program; Icelandic Heart Association: Hjartavernd; National Institute on Aging Intramural Research Program

**Keywords:** cognitive impairment, cardiovascular risk factor, age modification of risk, life-course

## Abstract

The mixed evidence that high levels of cardiovascular risk factors (CVRF) are associated with lower cognitive test scores of may be due to confounding of age across studies. We pooled and harmonized individual-level data (30,967 persons, age range 42–96y) from five prospective cohorts to examine the trajectories of betas estimating 1-year-age associations of a cognitive outcome (Digit Symbol Substitution Test; DSST) to five CVRF: systolic and e blood pressure, total cholesterol, fasting glucose and body mass index. Linear and quadratic piecewise regression models were fit to the trajectory patterns of these betas. The trajectories showed with each 1-year age increment, higher CVRF were associated with lower DSST, but associations attenuated toward zero as age increased. In addition, the pattern across age of each CVRF-DSST trajectory ranged from linear to non-liner. Without accounting for participant age in cohort comparisons, conclusions about the potential benefit on cognitive function of modifiable CVRF control will continue to be mixed and lead to delays in developing prevention programs.

## Introduction

Late-life cognitive disorders (CD), including cognitive impairment and dementia are devastating. Studies suggest the trajectory to CD begins decades before the onset of clinical symptoms, and that studies of risk factors require a life-course perspective (1). Research over the last two decades has raised the possibility that modifiable cardiovascular risk factors (CVRF) may increase the risk for late age CD, and therefore, their control may reduce the occurrence of disease. However, evidence for this hypothesis is mixed.

The inconsistent evidence is quantified by the variation in the slopes or risk ratios of the relationship between a CVRF (i.e., blood pressure) and CD outcomes. Reports from observational studies range from an expected higher CVRF level increasing the risk for CD, to a null association, to an unexpected lower CVRF level increasing the risk for CD.(2) The few clinical trials that test the effects on cognitive impairment of intervening on cardiovascular risk factors have largely been negative (3) (4) (5).

Reconciling these mixed findings is hampered because they are based on single studies of different aged cohorts (i.e. ≤55; ≥55, ≥65 or ≥75 yrs. of age); different study designs (i.e. cross-sectional, case-control or longitudinal); different target populations (i.e. clinical volunteers, patient groups, or population-based), different outcome measures (i.e. cognitive testing, clinical adjudication, or algorithmic) and follow-up time; as well as different statistical models to test for associations. Across cohorts, there may also be a different balance of confounding variables, which could bias not only the internal validity of the study, but also the validity of cross-cohort comparisons. It is also a challenge to study dementia prospectively given the long pre-clinical period. (6) Further the risk factor levels and variability may change with age; increasing brain pathology may alter the physiologic activity of a risk factor (leading to ‘reverse causation’) [1]; or declines in cognition may lead to less vigilant health care (7).

Age is a powerful moderating factor for risk factors, cognition and the emergence of disease leading to differences in CVRF-CD associations. To understand how the CVRF-CD associations may change over a broad age range we calculated 1-yr slopes of these associations for five CVRF and modeled the trajectory of these slopes over the age span. These 1-yr slopes were based on pooled data from five mid- and late-life cohorts that have measured the same cognitive test and set of CVRF.

## Methods

### Cohort descriptions and variable selection

#### Cohorts.

The following five cohorts of men and women were included in our synthetic cohort: Age Gene/Environment Susceptibility-Reykjavik Study (AGES-RS; baseline 2002/06, n=5764, 67–96 y, all European Caucasian,(8); Atherosclerosis Risk in Communities study (ARIC, 1987/89, baseline n=15,792, 44 to 64 y, bi-racial (9); Cardiovascular Health Study (CHS, baseline 1989, n=5888, 65–102 y, bi-racial (10); Coronary Artery Disease in Young Adults study (CARDA, baseline 1984/85, n=5115, 18–30y, bi-racial (11); and the Multi-ethnic Study of Atherosclerosis (MESA, baseline 2000/02, n= 6814, 44–84, 4 race/ethnicity groups.(12) The first cognitive tests in MESA, were acquired at the 5^th^ exam; CARDIA at the 25-year follow-up exam; AGES-RS at the baseline exam; ARIC at the 2^nd^ follow-up exam; and CHS at the baseline exam. The cohorts are briefly described in **Online Resource 1**.

#### Risk factors.

Five cardiovascular risk factors were selected for study: diastolic (DBP) and systolic (SBP) blood pressure (mmHG), body mass index (BMI, kg/m^2^), total cholesterol (mg/dL) and glucose (mg/dL). These factors are powerful predictors of CV disease and are part of the American Heart Association’s Simple 7 (13) markers of good cardiac health. These risk factors have been robustly measured in studies using standardized methods. Additionally, these CVRF in relation to CD have been systematically reviewed within the last five years (14),(15),(16),(17).

#### Cognitive outcome.

As dementia occurs only in later years with no events in middle age, we chose the Digit Symbol Substitution Test (DSST) (18) as the cognitive outcome. This test is informative in mid and late-life and predicts future, or reflects, current dementia (19–21). The DSST is an omnibus test of processing speed, visuospatial skills, and sustained attention, has an approximately normal distribution in mid and late-life individuals. (22),(23). The DSST is a standard paper-and-pencil based timed-test where the participant writes down symbols paired with a digit (133 pairings), as presented in a set of pairs given at the top of the page. However, time to complete the pairings differed by study (ARIC and CHS allowed participants 90 seconds for the test, and AGES-RS, CARDIA, and MESA allowed 120 seconds) thus changing the achievable maximum score. Additionally, the distribution and range of the DSST scores are influenced by cohort differences in age, sex, race/ethnicity, and education of the participants (24) (9). Therefore, we generated a harmonized DSST score that was comparable across cohorts (see below).

#### Covariates.

Because of their strong association with CVRF and DSST (9), the harmonization models included baseline age, sex, race/ethnicity (White vs. Other), education (≤ high school, high school, > high school); and smoking status (never smoked, past smoker, current smoker).

#### Analytical sample:

The harmonization sample was restricted to those with a complete *per exam* pairing of DSST and CVRF and with complete data on covariates. After filtering, the analytical sample included: AGES-RS, n=5,342, age range 66–96 yo; ARIC, n=13,698, 46–75 yo; CARDIA, n=3,334, 43–59 yo; and MESA, n= 4,059, 53–94 yo. Giving an analytical sample of 30,967 persons, age range 42–96 yo (Online resource Figures 2) sample size per 1-yr age bin).

### Statistical Methods

#### Data Harmonization.

Using the same method, CVRF and the DSST scores were harmonized to the MESA cohort as it had the widest age interval. The harmonization algorithm is described in detail in **Online Resource Text 3** and graphed in **Online resource Figures 4**. For example, for one risk factor (DBP) and one cohort (ARIC), the harmonized value of DBP was estimated as follows: First, a linear regression model ‘DBP= age, sex, education and smoking’ (as defined above) was estimated and the residuals (RijARIC) from that model were generated. These residuals reflect how much the individual differed from the cohort model prediction. Next, we ran a linear regression based on MESA data, whereby DBP is predicted by covariates. Then we weighted the ARIC covariates with the covariate betas generated from the MESA model, giving an ARIC/MESA-based prediction of DBP. The residuals from the ARIC (RijARIC) model were then added to the predicted ARIC-MESA DBP giving a harmonized DBP data point for ARIC. According to this method, ‘harmonized’ CVRF or DSST data points from MESA are identical to the original MESA data points. Comparisons of original and harmonized DSST and CVRF by cohort are illustrated in Online resource 5a-e and by age in Online resource 6).

#### Statistical models.

To estimate the trajectory of the slope (*T-slope*), data points (i.e. 1-yr slopes) from all harmonized cohorts were combined and each individual timepoint was binned by 1-yr age groups. To optimize sample size per age bin we combined the 1-yr slopes at the lower and upper age range (i.e. age bins ≤ 47 and ≥ 88), giving 42 age bins each with corresponding l CVRF-DSST 1-yr slope estimate.

The overall pattern of CVRF-DSST 1-yr slopes from middle to late age, the trajectory of slopes, T-slope, was calculated by modeling the CVRF-DSST 1-yr-slope (Y) as a linear function of the age in each age bin (X). Upon inspection of graphical data and confirmed by the poor fit of the linear model [model 1] it was evident that, with age, some 1-yr slopes began to increase or decrease for some CVRF-DSST slopes. This suggests the association between a CVRF and DSST changes with age, as described above. Based on the patterns of the trajectories of slopes, we sought to define the best-fitting model. To do this we compared linear, and piecewise linear and quadratic models using the Bayesian information criterion (BIC; MR0468014) (see Online resource Tables 7).

The linear-linear piecewise model estimated each of the two *T-slopes* (i.e., pieces) as a linear function. The second piecewise model, linear-quadratic, estimated one piece as a linear function, and the second piece as a quadratic function. To decide where to cut the model into two pieces, we estimated *agecut*, the age at which BIC was the lowest, generally resulting in a strong shift in the direction of the *T-slope* (see model details in the Supplemental Materials). For all three models, each data point (CVRF-DSST 1-yr-slopes per corresponding age bin) was weighted by the inverse of the square of the standard error of the CVRF-DSST 1-yr-slope (See **Online Resource Text 8** for details on the model development).

There is some dependency between age bins since some individuals in a 1-yr age bin can also have a timepoint in another older 1-yr age bin which, if not accounted for, would lead to smaller, and thus overly optimistic *p*-values and confidence limits. To correct for age-bin dependency, we performed 1000 iterations using sampling with replacement (bootstrap) on our initial dataset. For each iteration, we saved the piecewise model parameter estimates to estimate the standard error of those estimates to generate a correct z-score and adjust the p-values and confidence intervals accordingly.

We verified the model fit by visually comparing the trajectory estimated by the model to a smoothed trajectory based on a procedure with a triangular smoothing window of size 11 (Online Resource Text 9 describes the smoothing algorithms) and that accounts for the variance of the 1-yr-slopes and number of cases per age bin.

### Analytical questions

Based on these models we addressed the following questions: Across mid- and late-life: 1. are the 1-yr slopes < 0? (suggesting the 1-yr slopes are negative such that as the level of the CVRF increases, the DSST score decreases) 2. Is the overall trajectory of slopes constant? (i.e., is *T-slope* = 0, suggesting the association of CVRF to DSST is the same at all ages) 3. If the association of CVRF to DSST is not the same for linear models, or within each of the two pieces of the piecewise models, do the 1-yr-bin slopes increase or decrease? (suggesting the association of the CVRF to DSST changes with age).

## Results

**Figure 1:**
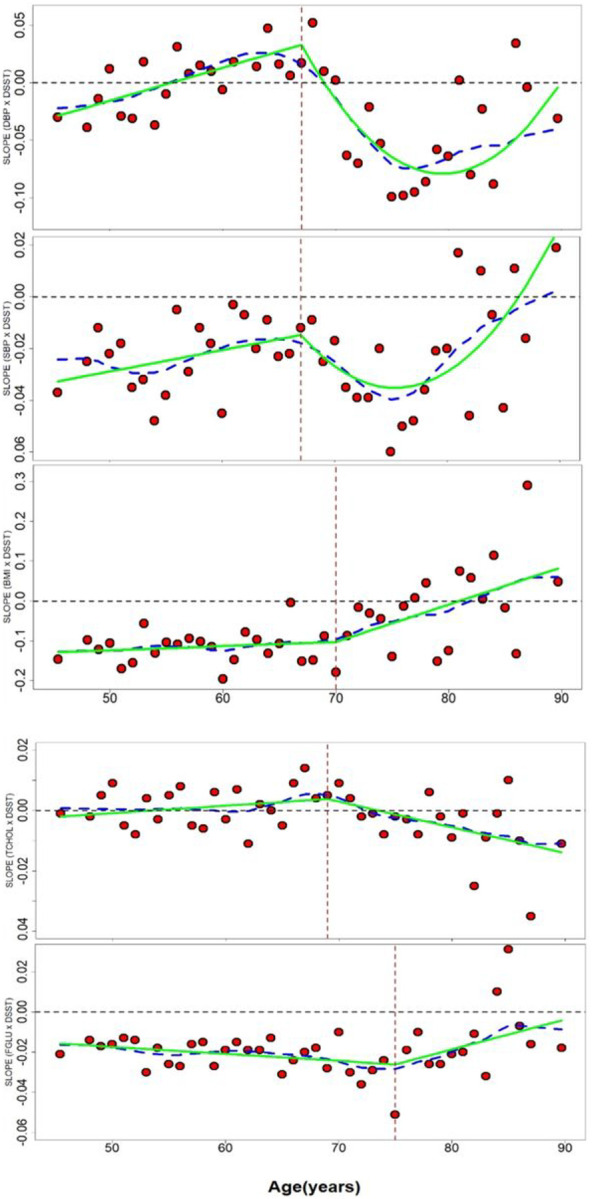
1a-1e: Five Cardiovascular risk factor – DSST relationships by 1-yr age bins Each dot represents the magnitude and direction of the 1-yr association between the risk factor and the DSST cognitive score. The vertical stippled line represents the age at which the slope of the betas significantly changed (i.e. Figure for BMI the line is at age 70 yrs.). The horizonal line at the y-axis zero is the point above which yearly slopes increase and below which decreases. The stippled line over the age represents the smoothed trajectory and the green line represents the modeled trajectory. Specific risk factors as modeled (2 spline components): 1a. DBP – linear-quadratic; 2a. SBP – linear-quadratic; 3a. BMI – linear-linear; 4a. cholesterol – linear-linear; 5a. Fasting glucose - linear-linear. See model parameters in the text.

The percent women was generally similar across cohorts (range 42.1% to 46.8%). At baseline (Table 1), ARIC and CARDIA had the youngest participants and AGES-RS the oldest. Trends in CVRF and DSST across cohorts generally tracked with relative ages of the cohorts. The mean and standard deviation of harmonized and unharmonized variables are shown in Online Resource Tables 10. The mean of the observed DSST-CVRF 1-yr slope per age bin, the modelled trajectory of the 1-yr slopes and the smoothed trajectory of the 1-yr slopes are shown in Fig 1a–e.

The linear-quadratic model for DBP (Fig 1a) had the lowest BIC, with *agecut*=67. Over the age span, there was considerable variation in the 1-yr slopes. For younger ages (less than 55 y) and older ages (≥67 y) 1-yr slopes were mostly negative (i.e., < 0), suggesting higher DBP is associated with lower DSST scores. Between 55 yo and 70 yo, 1-yr slopes attenuated and became positive close to zero (p = 5.3E-3). Although still positive at age 67, after this, slopes start a downward trend and become increasingly negative until approximately 80 y of age, when again the 1-yr slopes increasingly attenuated to zero (Tslope2sq = 0.00072 (0.00045,0.00099; p = 2.4E-7). This translates into an estimated 0.013 higher DSST score at age 60 y; a 0.21 lower DSST score at age 70 yo; and a 0.27 lower DSST score at age 80 yo for each increment in 1 mmHg DBP, controlling for covariates.

Similar to DBP, the SBP (Figure 1b) linear-quadratic model had the lowest BIC, with *agecut*=67 y. Individual 1-yr SBP-DSST-slopes were negative across the age span but became increasingly negative as the age, or relative minimum (nadir), of 75.4 yo was reached. This pattern suggests a higher SBP is associated with an even lower DSST score as age increased. After age 75 yo, the 1-yr slopes become increasingly less negative with age. Together these changes reflect a U-shaped relationship from age 67 to 88+ years old [T-slope2sq = 0.00029 (0.00014,0.00044; p = 1.3E-4)]. Based on this model, with each increment in 1 mmHg SBP, adjusting for covariates, there is an estimated 0.021 lower DSST score at age 60 yo; a 0.085 lower DSST at age 70 yo; and at age 80 yo there is a 0.085 lower DSST score.

The BMI trajectory was best modeled (lowest BIC) by the linear-linear model (Figure 1c) analysis, with *agecut* = 70 yo. For age ≤ 70, for each unit increase in the 1-yr slopes of DSST-BMI, there was an average 0.12 lower DSST; however, the trajectory is not significant (p=0.5) and approximately constant. After age 70 yo, 1-yr-slopes become increasingly less negative with age (p=1.5E-3), attenuating to zero and becoming positive slopes around age 80 yo, i.e., a higher BMI was associated with a higher DSST score after 80 years of age. This translates into for each increment of 1 BMI unit, there is an estimated 0.11 lower DSST score at age 60 yo; a 0.10 lower DSST score at age 70 yo; and a 0.009 lower DSST score at age 80 yo.

The best fitting model for total cholesterol was the linear-linear model (Figure 1d) and *agecut* = 69. Overall, 1-yr slopes were constant and close to zero for age < 69 yo (p = 0.29). After age 69 yo, the magnitude of the 1-yr-slopes of the relationship between DSST and cholesterol tended towards more negative such that a higher cholesterol was associated with a lower DSST score (p=2.2E-3). This translates to an estimated 0.0016 higher DSST score with each increment in 1 mg/dL of cholesterol at age 60 yo; a 0.0029 higher DSST score at age 70 yo; and 0.0056 lower DSST score at age 80 yo.

For fasting glucose, the linear-linear model for fasting glucose (Figure 1e), had the lowest BIC, with *agecut* = 75 yo. Overall, 1-yr slopes for DSST-fasting glucose associations were negative, meaning that for a unit increase in fasting glucose, there was a lower DSST score. The linear trends before (p=0.07) and after (p=0.057) age 75 were relatively constant but attenuate to zero. For age ≤ 75 yo, the expected 1-yr-slopes were marginally lower by 0.00035 for each increase in 1-unit of fasting glucose. After age 75 yo, the expected 1-yr-slopes was 0.0015 points higher with each 1-unit increment of age, with a drift of the 1-yr slopes attenuating towards zero. This translates into an estimated 0.021 lower DSST score at age 60 yo; a 0.024 lower DSST score at age 70 yo; and a 0.019 lower DSST score at age 80 yo for each increment in 1 unit of fasting glucose level.

## Discussion

In 2018, the National Academy of Science summit on prevention of cognitive decline [Preventing Cognitive Decline and Dementia: A Way Forward, National Academies Press, http://www.nap.edu; and https://www.nia.nih.gov/research/administration/recommendations-nih-ad-research-summit-2018] concluded the evidence for control of CVRF to reduce the risk for CD is not robust or consistent enough to support prevention trials or for public health messaging. Therefore to move forward a better understanding of factors underlying the incongruent results.

One source of variability in the evidence may be the study design (cross-sectional vs longitudinal), the age of individual study participants and the mean age of study cohorts. To control for study design we included in our pooled analyses only longitudinal community-based studies that used similar methods to measure cognitive function and key risk factors for cognitive decline. We found, controlling for study design and major confounders of sex, race, education and smoking, the five CBRF we studied were *negatively* associated with DSST test scores in both middle- and late-age. However, the magnitude and direction of the CVRF-DSST slope changed with age. All the CVRF 1-year slopes attenuated towards zero as age increased. There were CVRF differences in the age at which the direction of slopes was estimated to change, but generally slopes began to change between the late 60s and mid 70s age-band.

This analysis does not address the specific reasons for the direction and magnitude of changes in slope trajectories, although other studies can lend insight into contributing factors. Reverse causality has often been proffered as a ‘biologic’ explanation for finding unexpected (i.e., positive) directionality of associations. Biologically, reverse causality could result from a change in the balance between the brain and periphery regulation of CVRF, whereby the pathology in the brain regulates the risk factor, and not vice versa, as hypothesized. For instance, neurodegenerative pathology in the hypothalamus may affect regulation of glucose, insulin or body weight (25). These changes may result in observations that people on course to develop dementia are losing weight faster and their blood pressure and cholesterol levels are dropping faster than older persons who maintain cognitive function levels (26) (27). However, data are limited on the empirical significance these changes have on estimates of the relationships between risk factors and cognition (28).

Although 1-yr CVRF-DSST slopes were negative, several were close to zero, especially at older ages, suggesting other factors may explain null to positive associations between CVRF and DSST. A change in the magnitude or direction of 1-yr slopes may reflect increasing co-morbidity as people age and the proportional reduction in how much any one risk factor can explain CD; the CVRF may lose predictive power with age; or there may be biases due to dropout or mortality [29].

Interestingly, the BP – DSST relationships were the most variable. High blood pressure is a strong risk factor for underlying brain pathology that can lead to CD (30) and possibly, among the five CVRF we studied, the most direct measure of a factor causing cerebral damage. BP may also be the most sensitive to differences in measurement techniques, timing of measurement and individual/cohort differences such as the prevalence of anti-hypertension use. At the same time, compared to the other risk factors we studied, high BP is likely a stronger risk factor for death at later ages. The more variable results in the DBP compared with SBP analysis is consistent with the epidemiology of blood pressure. With aging, SBP tends to increase, while DBP may decrease due to vascular disease such as increased stiffness (31).

Age is the strongest risk factor for chronic disease, and particularly for CD. Our study suggests comparative analysis of the observational study evidence for CVRF-cognitive associations should be reported and interpreted at least taking into account the age range of the cohort. Specific studies with a wide age interval should qualitatively or quantitatively examine age interactions. Additional modelling of the reverse causation confound could also contribute to better understanding of how CV risk factors interact with dementing pathophysiology (28, 32) (33).

This study has implications for clinical trials aimed to reduce cognitive decline by treating cardiovascular RF. Observational studies, particularly those based on longitudinal well-characterized cohorts can be pooled and harmonized, and can point to candidate CVRF, and to time intervals when the impact of an intervention is detectable. However the effect sizes are likely small so clinical trials conducted at later ages will need large sample sizes or target vulnerable sub-groups and will need to deliver an intervention that will create large differences between treatment arms, such as SPRINT MIND, where the difference in SBP in the treatment vs control arms was SBP > 14 mmHg (3).

Our analysis has several strengths. We selected cohorts with a similar prospective cohort design and examined core CVRF that have been standardized across studies and time. We also chose one cognitive test that was collected by all studies (34) and is informative in mid and late age. Differences in the distribution of confounding factors were reduced by using common risk factors to adjust the models. Our harmonization algorithm across cohorts successfully standardized measures to one cohort; and by harmonizing the intercepts we aligned the linear slope estimates across cohorts. We also identified the ‘best fitting’ linear and non-linear slope trajectory models by comparing Bayesian Information Criteria (BIC) across linear, linear-linear and linear-quadratic equations.

However, several aspects of our study should be noted when interpreting the results. DSST scores were not imputed thus minimizing that source of error. However, because the exam at which a cohort introduced cognitive testing varied, we had to exclude the younger individuals in CARDIA and ARIC. It should also be noted that the trajectories are based on individual 1-yr slopes of CVRF--DSST associations, which are cross-sectional and do not address our question within individual CVRF trajectories. Therefore, while pooling provided a larger sample and a more cohesive picture of differences in CVRF-DSST associations over time, this cross-sectional analysis is subject to the same biases as other cross-sectional studies of ‘change by age’– most importantly the bias caused by mortality or selective dropout by age and health condition. We used a single cognitive test with a normal distribution of test scores reflecting psychomotor speed and executive function; tests with different properties may give different results. We did not explore the life-course pattern of CVRF in relation to a dementia outcome, which requires additional assumptions, harmonizing dementia diagnoses and developing prediction models for younger cohorts with no dementia outcomes.

In conclusion, understanding the methodologic and demographic differences among observational studies will aid in the interpretation of CVRF – CD associations and promote more targeted design of clinical trials to prevent cognitive loss in late-life.

## Figures and Tables

**Table 1:** Description of all participants with harmonized and complete data in pooled analysis by cohort

	AGES	ARIC	CARDIA	CHS	MESA
N persons	5,342	13,698	3,334	4,534	4,059
N observations	8,299	26,028	3,334	6,853	4,059
Age y (mean/range)	77.7 (66–96)	59.8 (46–75)	50.2 (42–59)	74.4 (63–95)	69.4 (53–94)
Sex (% females)	42.3	44.5	43.6	42.1	46.8
Diastolic BP (mmHg)^a^	65.4 (10.3)	69.4 (10.3)	71.8 (10.9)	66.1 (12.8)	68.5 (10.1)
Systolic BP (mmHg)^a^	126.4 (20.7)	116.9 (18.6)	111.6 (15.3)	124.4 (21.7)	123.8 (20.7)
Body mass index^a^	27.3 (4.2)	29.2 (5.4)	29.6 (7.0)	27.6 (4.2)	28.4 (5.6)
Total Cholesterol^a^ (mg/dL)	178.2 (44.3)	187.8 (39.9)	195.5 (37.9)	180.6 (39.2)	183.3 (37.1)
Fasting Glucose^a^ (mg/dL)	98.8 (21.2)	101.5 (40.6)	101.4 (28.5)	98.9 (31.9)	102.0 (28.7)
DSST^a^ (no. correct)	45.3 (12.3)	58.4 (14.1)	69.7 (15.3)	48.4 (14.0)	50.6 (18.3)
Smoking (%current)	10.8	19	18.7	7.8	7.8
Education (%<12 yrs.)	19.6	20.3	1.7	23.7	14
White (%)^b^	100	76.7	53.7	95.8	40.4

a mean (SD)

b White compared to Non-white participants

CHS: The Cardiovascular Health Study, ARIC: Atherosclerosis Risk in the Communities Study, MESA: the Multi-Ethnic Study of Atherosclerosis; CARDIA: the Coronary Artery Risk Development in Young Adults; AGES-RS: The Age Gene/Environment Susceptibility-Reykjavik Study; DSST: Digit Symbol Substitution Test

## Data Availability

Data from ARIC, CARDIA, CHS and MESA, were accessed from the NHLBI Biologic Specimen and Data Repository Information Coordinating Center [https://biolincc.nhlbi.nih.gov/studies]. Data from the AGES-RS study are available at the Icelandic Heart Association upon request [https://hjarta.is/en/]. The derived data generated in this research will be shared on reasonable request to the corresponding author (launerl@nia.nih.gov).

## References

[1] Launer LJ. The epidemiologic study of dementia: a life-long quest? Neurobiol Aging. 2005;26(3):335–40. doi:10.1016/j.neurobiolaging.2004.03.016 15639311

[2] Livingston G, Huntley J, Sommerlad A, Dementia prevention, intervention, and care: 2020 report of the Lancet Commission. Lancet. 2020;396(10248):413–46. doi:10.1016/S0140-6736(20)30367-6 32738937 PMC7392084

[3] Group SMIftSR, Williamson JD, Pajewski NM, Effect of Intensive vs Standard Blood Pressure Control on Probable Dementia: A Randomized Clinical Trial. JAMA. 2019;321(6):553–61. doi:10.1001/jama.2018.21442 30688979 PMC6439590

[4] Launer LJ, Miller ME, Williamson JD, Effects of intensive glucose lowering on brain structure and function in people with type 2 diabetes (ACCORD MIND): a randomised open-label substudy. Lancet Neurol. 2011;10(11):969–77. doi:10.1016/S1474-4422(11)70188-0 21958949 PMC3333485

[5] Sink KM, Espeland MA, Castro CM, Effect of a 24-Month Physical Activity Intervention vs Health Education on Cognitive Outcomes in Sedentary Older Adults: The LIFE Randomized Trial. JAMA. 2015;314(8):781–90. doi:10.1001/jama.2015.961 26305648 PMC4698980

[6] Jack CR Jr., Bennett DA, Blennow K, NIA-AA Research Framework: Toward a biological definition of Alzheimer’s disease. Alzheimers Dement. 2018;14(4):535–62. doi:10.1016/j.jalz.2018.02.018 29653606 PMC5958625

[7] Punthakee Z, Miller ME, Launer LJ, Poor cognitive function and risk of severe hypoglycemia in type 2 diabetes: post hoc epidemiologic analysis of the ACCORD trial. Diabetes Care. 2012;35(4):787–93. doi:10.2337/dc11-1855 22374637 PMC3308284

[8] Harris TB, Launer LJ, Eiriksdottir G, Age, Gene/Environment Susceptibility-Reykjavik Study: multidisciplinary applied phenomics. Am J Epidemiol. 2007;165(9):1076–87. doi:10.1093/aje/kwk115 17351290 PMC2723948

[9] Knopman DS, Mosley TH, Catellier DJ, Coker LH, Atherosclerosis Risk in Communities Study Brain MRIS. Fourteen-year longitudinal study of vascular risk factors, APOE genotype, and cognition: the ARIC MRI Study. Alzheimers Dement. 2009;5(3):207–14. doi:10.1016/j.jalz.2009.01.027 19362884

[10] Kuller LH, Lopez OL, Newman A, Risk factors for dementia in the cardiovascular health cognition study. Neuroepidemiology. 2003;22(1):13–22. doi:10.1159/000067109 12566949

[11] Reis JP, Loria CM, Launer LJ, Cardiovascular health through young adulthood and cognitive functioning in midlife. Ann Neurol. 2013;73(2):170–9. doi:10.1002/ana.23836 23443990 PMC3608821

[12] Moazzami K, Ostovaneh MR, Ambale Venkatesh B, Left Ventricular Hypertrophy and Remodeling and Risk of Cognitive Impairment and Dementia: MESA (Multi-Ethnic Study of Atherosclerosis). Hypertension. 2018;71(3):429–36. doi:10.1161/HYPERTENSIONAHA.117.10289 29378853 PMC5812794

[13] Lloyd-Jones DM, Hong Y, Labarthe D, Defining and setting national goals for cardiovascular health promotion and disease reduction: the American Heart Association’s strategic Impact Goal through 2020 and beyond. Circulation. 2010;121(4):586–613. doi:10.1161/CIRCULATIONAHA.109.192703 20089546

[14] Anstey KJ, Ashby-Mitchell K, Peters R. Updating the Evidence on the Association between Serum Cholesterol and Risk of Late-Life Dementia: Review and Meta-Analysis. J Alzheimers Dis. 2017;56(1):215–28. doi:10.3233/JAD-160826 27911314 PMC5240556

[15] Geijselaers SLC, Sep SJS, Stehouwer CDA, Biessels GJ. Glucose regulation, cognition, and brain MRI in type 2 diabetes: a systematic review. Lancet Diabetes Endocrinol. 2015;3(1):75–89. doi:10.1016/S2213-8587(14)70148-2 25163604

[16] Albanese E, Launer LJ, Egger M, Body mass index in midlife and dementia: Systematic review and meta-regression analysis of 589,649 men and women followed in longitudinal studies. Alzheimers Dement (Amst). 2017;8:165–78. doi:10.1016/j.dadm.2017.05.007 28761927 PMC5520956

[17] Qiu C, Winblad B, Fratiglioni L. The age-dependent relation of blood pressure to cognitive function and dementia. Lancet Neurol. 2005;4(8):487–99. doi:10.1016/S1474-4422(05)70141-1 16033691

[18] Jaeger J. Digit Symbol Substitution Test: The Case for Sensitivity Over Specificity in Neuropsychological Testing. J Clin Psychopharmacol. 2018;38(5):513–9. doi:10.1097/JCP.0000000000000941 30124583 PMC6291255

[19] Brody DJ, Kramarow EA, Taylor CA, McGuire LC. Cognitive Performance in Adults Aged 60 and Over: National Health and Nutrition Examination Survey, 2011–2014. Natl Health Stat Report. 2019(126):1–23. 31751207

[20] Schneider AL, Gottesman RF, Mosley T, Cognition and incident dementia hospitalization: results from the atherosclerosis risk in communities study. Neuroepidemiology. 2013;40(2):117–24. doi:10.1159/000342308 23095770 PMC3642775

[21] Sun D, Thomas EA, Launer LJ, Sidney S, Yaffe K, Fornage M. Association of blood pressure with cognitive function at midlife: a Mendelian randomization study. BMC Med Genomics. 2020;13(1):121. doi:10.1186/s12920-020-00769-y 32847530 PMC7448985

[22] Amieva H, Meillon C, Proust-Lima C, Dartigues JF. Is Low Psychomotor Speed a Marker of Brain Vulnerability in Late Life? Digit Symbol Substitution Test in the Prediction of Alzheimer, Parkinson, Stroke, Disability, and Depression. Dement Geriatr Cogn Disord. 2019;47(4–6):297–305. doi:10.1159/000500597 31466055

[23] Liao H, Zhu Z, Wang H, Rong X, Young CA, Peng Y. Cognitive Performance Concomitant With Vision Acuity Predicts 13-Year Risk for Mortality. Front Aging Neurosci. 2019;11:65. doi:10.3389/fnagi.2019.00065 30967772 PMC6439522

[24] Fitzpatrick AL, Rapp SR, Luchsinger J, Sociodemographic Correlates of Cognition in the Multi-Ethnic Study of Atherosclerosis (MESA). Am J Geriatr Psychiatry. 2015;23(7):684–97. doi:10.1016/j.jagp.2015.01.003 25704999 PMC4465027

[25] Kullmann S, Kleinridders A, Small DM, Central nervous pathways of insulin action in the control of metabolism and food intake. Lancet Diabetes Endocrinol. 2020;8(6):524–34. doi:10.1016/S2213-8587(20)30113-3 32445739

[26] Wagner M, Helmer C, Tzourio C, Berr C, Proust-Lima C, Samieri C. Evaluation of the Concurrent Trajectories of Cardiometabolic Risk Factors in the 14 Years Before Dementia. JAMA Psychiatry. 2018;75(10):1033–42. doi:10.1001/jamapsychiatry.2018.2004 30043038 PMC6233804

[27] Stewart R, White LR, Xue QL, Launer LJ. Twenty-six-year change in total cholesterol levels and incident dementia: the Honolulu-Asia Aging Study. Arch Neurol. 2007;64(1):103–7. doi:10.1001/archneur.64.1.103 17210816

[28] Flanders WD, Augestad LB. Adjusting for reverse causality in the relationship between obesity and mortality. Int J Obes (Lond). 2008;32 Suppl 3:S42–6. doi:10.1038/ijo.2008.84 18695652

[29] Rouanet A, Avila-Rieger J, Dugravot A, How Selection Over Time Contributes to the Inconsistency of the Association Between Sex/Gender and Cognitive Decline Across Cognitive Aging Cohorts. Am J Epidemiol. 2022 Feb 19;191(3):441–452. doi: 10.1093/aje/kwab227. 34521111 PMC9214252

[30] Iadecola C, Yaffe K, Biller J, Impact of Hypertension on Cognitive Function: A Scientific Statement From the American Heart Association. Hypertension. 2016;68(6):e67–e94. doi:10.1161/HYP.0000000000000053 27977393 PMC5361411

[31] Rahimi K, Emdin CA, MacMahon S. The epidemiology of blood pressure and its worldwide management. Circ Res. 2015;116(6):925–36. doi:10.1161/CIRCRESAHA.116.304723 25767281

[32] Spearing NM, Connelly LB, Nghiem HS, Pobereskin L. Research on injury compensation and health outcomes: ignoring the problem of reverse causality led to a biased conclusion. J Clin Epidemiol. 2012;65(11):1219–26. doi:10.1016/j.jclinepi.2012.05.012 23017639

[33] Kivimaki M, Singh-Manoux A, Pentti J, Physical inactivity, cardiometabolic disease, and risk of dementia: an individual-participant meta-analysis. BMJ. 2019;365:l1495. doi:10.1136/bmj.l1495 30995986 PMC6468884

[34] Briceño EM, Gross AL, Giordani BJ, Manly JJ, Gottesman RF, Elkind MSV, Sidney S, Hingtgen S, Sacco RL, Wright CB, Fitzpatrick A, Fohner AE, Mosley TH, Yaffe K, Levine DA. Pre-Statistical Considerations for Harmonization of Cognitive Instruments: Harmonization of ARIC, CARDIA, CHS, FHS, MESA, and NOMAS. J Alzheimers Dis. 2021;83(4):1803–1813. doi: 10.3233/JAD-210459. 34459397 PMC8733857

